# Evaluating Cardiovascular Disease (CVD) risk scores for participants with known CVD and non-CVD in a multiracial/ethnic Caribbean sample

**DOI:** 10.7717/peerj.8232

**Published:** 2020-03-09

**Authors:** Amalia Hosein, Valerie Stoute, Samantha Chadee, Natasha Ramroop Singh

**Affiliations:** 1 Biomedical Engineering, The University of Trinidad and Tobago, O’Meara, Arima, Trinidad & Tobago; 2 Environmental Studies, The University of Trinidad and Tobago, O’Meara, Arima, Trinidad & Tobago

**Keywords:** Cardiovascular diseases, Risk prediction models, QRISK^®^2, Framingham, ASSIGN, Caribbeans, Multiracial, Multiethnic

## Abstract

**Background:**

Cardiovascular Disease (CVD) risk prediction models have been useful in estimating if individuals are at low, intermediate, or high risk, of experiencing a CVD event within some established time frame, usually 10 years. Central to this is the concern in Trinidad and Tobago of using pre-existing CVD risk prediction methods, based on populations in the developed world (e.g. ASSIGN, Framingham and QRISK^®^2), to establish risk for its multiracial/ethnic Caribbean population. The aim of this study was to determine which pre-existing CVD risk method is best suited for predicting CVD risk for individuals in this population.

**Method:**

A survey was completed by 778 participants, 526 persons with no prior CVD, and 252 who previously reported a CVD event. Lifestyle and biometric data was collected from non-CVD participants, while for CVD participants, medical records were used to collect data at the first instance of CVD. The performances of three CVD risk prediction models (ASSIGN, Framingham and QRISK^®^2) were evaluated using their calculated risk scores.

**Results:**

All three models (ASSIGN, Framingham and QRISK^®^2) identified less than 62% of cases (CVD participants) with a high proportion of false-positive predictions to true predictions as can be seen by positive predictabilities ranging from 78% (ASSIGN and Framingham) to 87% (QRISK^®^2). Further, for all three models, individuals whose scores fell into the misclassification range were 2X more likely to be individuals who had experienced a prior CVD event as opposed to healthy individuals.

**Conclusion:**

The ASSIGN, Framingham and QRISK^®^2 models should be utilised with caution on a Trinidad and Tobago population of intermediate and high risk for CVD since these models were found to have underestimated the risk for individuals with CVD up to 2.5 times more often than they overestimated the risk for healthy persons.

## Introduction

Cardiovascular Disease is the largest contributor to mortality and morbidity worldwide accounting for 31% of all global deaths (CSO, 2011; WHO, 2016a, 2016b, 2017). Of these deaths, 85% are due to heart attack and stroke. The Caribbean region is no exception (Francis et al., 2015) with a mean of 32% (26–36%) of total mortality per year from CVD (CSO, 2011; WHO, 2016a). The decision about whether to initiate specific preventive action and to what extent is mainly guided by the estimation of risk for such a vascular event occurring in an individual (Payne, 2012).

A population-based approach to CVD risk scores is advantageous since existing risk score estimates seem to perform poorly in the developing countries and may lead to misclassification of individuals who do and do not require treatments (Chamnan & Aekplakorn, 2017). Whilst many developing countries solely describe estimated cardiovascular risk by applying existing CVD risk scores to their populations’ cross-sectional data, a number of countries have validated and recalibrated existing risk scores and only a few have developed new risk scores specific to their populations (Yamwong, 2005; Wu et al., 2006; Assmann, Cullen & Schulte, 1998).

The risk prediction charts which accompany these guidelines allow treatment to be targeted according to simple predictions of absolute cardiovascular risk (Sisa, 2018; Liew et al., 2018). The modifiable (cholesterol, weight, blood pressure, among others) and non-modifiable (usually age, sex, family history) risk factors have been used to create risk prediction algorithms in order to estimate the 10-year risk of having a CVD event. Three such CVD risk models are the Framingham risk score (Wilson et al., 1998), the ASSIGN score (Tunstall-Pedoe, 2005), and the QRISK^®^2 score (Hippisley-Cox et al., 2007). The Framingham risk score is based on a US cohort recruited several decades ago (Kannel, McGee & Gordon, 1976). The ASSIGN risk score was derived from the Scottish Heart Health Extended Cohort (De La Iglesia et al., 2011) and the QRISK^®^2 risk score from a large primary care database in England and Wales (Hippisley-Cox et al., 2008). All three models determine the % risk of an individual experiencing a CVD event in the next 10 years. Individuals estimated as having <10%, 10–20%, and >20% CVD risk are considered to have low, intermediate, and high risk, respectively, of having a CVD event in the next 10 years.

These epidemiologic risk models, however, may not always account for variations which exist among regions and countries due to different lifestyles, socio-economic conditions, and genetic predispositions (Sisa, 2018; Liew et al., 2018; Siontis et al., 2012). Clinicians, however, still utilise the risk models to guide their diagnostics (Sisa, 2018), even though it is recognised that risk models may perform differently in populations of different racial or ethnic backgrounds (D’Agostino et al., 2001; Liu et al., 2004). Thus, systematic efforts for model validation in other populations is essential. In addition, no estimation of the most sensitive CVD risk prediction model for a Caribbean population with significant African and East Indian ethnic sub-populations, has yet been made.

Ethnicity has been well established worldwide and in the Caribbean as a CVD risk factor (Hippisley-Cox et al., 2007; Martin, 2009; Beckles et al., 1986; Ezenwaka, Premanand & Orrett, 2000). In Trinidad and Tobago, the ethnic composition is 34.2% Africans, 35.4% East Indians, 23% Mixed (all races of which 7.7% is Indo/Afro) and 7.5% Other ethnicity (Chinese, Syrian, White) (CSO, 2011). Population statistics are for adult individuals residing in Trinidad and Tobago more than 15 years (or for children all of their lives). Afro-Trinbagonians are designated as those who trace their heritage to Africa in the period since Christopher Columbus’s arrival in the region in 1492. Indo-Trinbagonians (or East Indians) trace their heritage to India and are mostly descendants of the original indentured workers brought by the British, the Dutch and the French from 1845. Mixed-Trinbagonians are those participants with roots in Africa, India, Europe, Venezuela, and several other countries.

This article evaluates the validity of three existing CVD risk models in establishing individual CVD risk scores for a multiracial/ethnic Caribbean population. Risk factors from a sample of Trinidad and Tobago participants, of known CVD status, were used to calculate their risk scores for each of three existing CVD risk prediction models—QRISK^®^2, Framingham, and ASSIGN. The efficacies of these models, for correctly classifying the status of individuals in the sample, are compared. The focus is also on the misclassification errors for the three models, with particular attention paid, for each model, to the relative misclassification for CVD versus non-CVD individuals in this multiracial/ethnic Caribbean sample.

## Materials and Methods

### Study sample (viz. TT2015 dataset: DOI 10.6084/m9.figshare.7752440)

**Figure 1:**
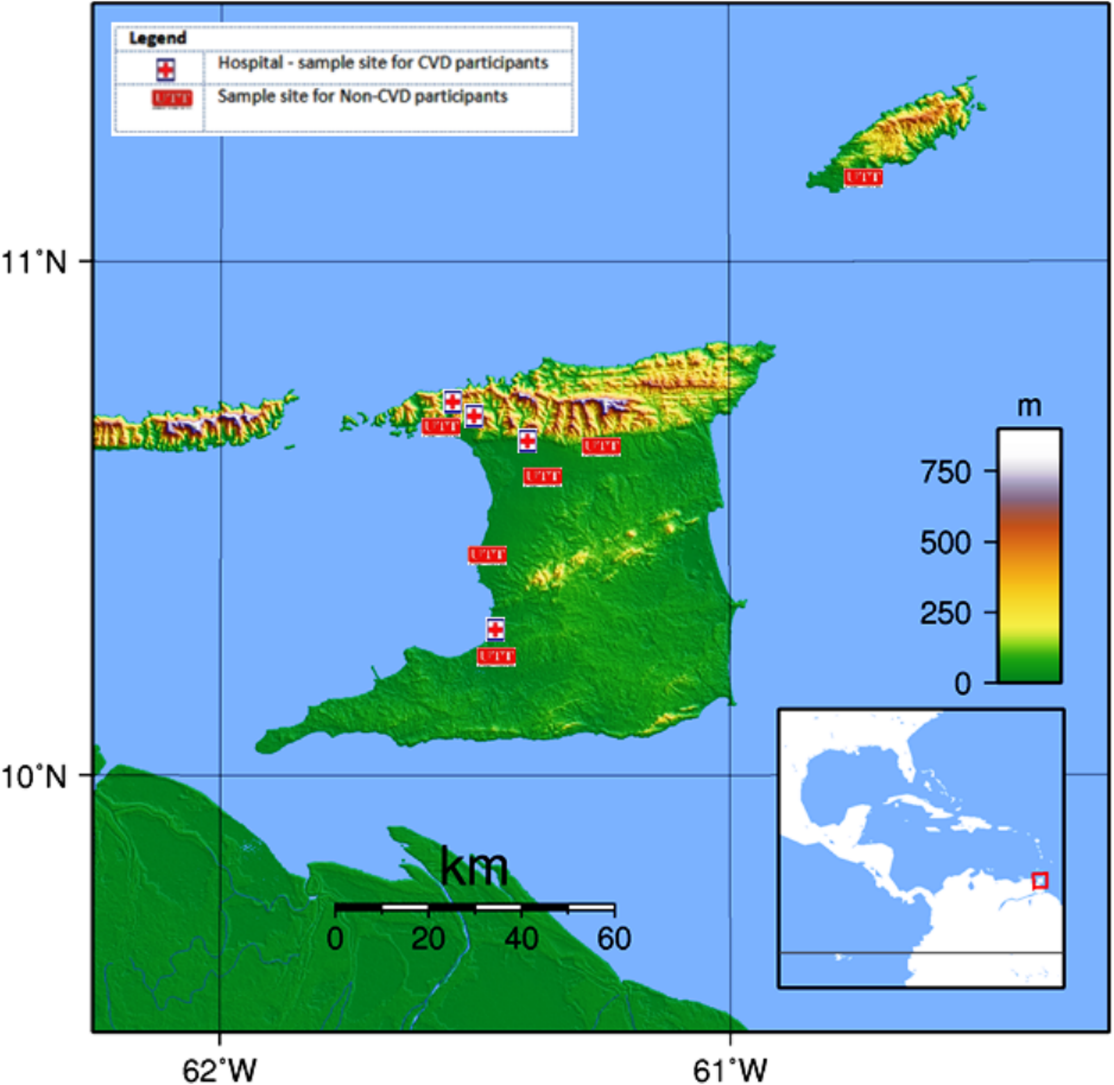
Map of Trinidad and Tobago showing the UTT sample sites for non-CVD participants and hospitals for CVD participants. Map credit: Wikimedia, 2007. Licensed under CC BY 3.0 SA.

Information was collected from a group of 791 participants between the ages of 18 and 75 during the period October 2013 to May 2014. Thirteen (13) individuals were excluded due to missing data. Of the 778 remaining participants, 526 were from the general public and had never previously experienced a CVD event (designated as non-CVD) and 252 others, who had been previously diagnosed with CVD, were obtained from three general hospitals and one private hospital, all located in Trinidad (viz. San Fernando General Hospital, Port-of-Spain General Hospital, Eric Williams Medical Sciences Complex, and Doctor’s Inn Private Hospital). For the purpose of this study, CVD patients were defined as those with any disorder of the heart or blood vessels and included those who had experienced an acute myocardial infarction (MI), silent MI, coronary surgery, and/or strokes or who currently had atherosclerosis and/or stent implants (De Fatima Marinho De Souza et al., 2012; Whitehead, Ford & Gama, 2014; CSO, 2011; WHO, 2016a, 2016b). Pregnant women and persons who had not resided in Trinidad and Tobago for the last 20 years were excluded. Non-CVD participants were recruited using flyers and they were tested at six locations throughout the country (Fig. 1).

### Data collection

This study received the requisite ethical approvals from The University of Trinidad and Tobago Internal Review Board, the Ethics Committee of the Ministry of Health of Trinidad and Tobago and the North-Central Regional Health Authority. All participants were presented with an overview of the study and the study requirements and provided their signature as an acknowledgment of their voluntary participation.

For non-CVD participants a questionnaire was administered to collect information on age, sex, ethnicity, smoking habits, family history of CVD, diet, and lifestyle. Measurements for weight, height, waist and hip circumference, blood pressure, and lipid profiles (POC Cardiochek PA analyser from PTS Diagnostics, USA (Matteucci et al., 2014; Panz et al., 2005)) were recorded for each participant.

For CVD participants, relevant data was collected from their hospital medical records at the first instance of a physician-diagnosed CVD event, which included any disorder of the heart or blood vessels and included those who had experienced an acute myocardial infarction (MI), silent MI, coronary surgery, and/or strokes or who currently had atherosclerosis and/or stent implants (De Fatima Marinho De Souza et al., 2012; Whitehead, Ford & Gama, 2014; CSO, 2011; WHO, 2016a, 2016b). Data collected included their age, lipid profiles, blood pressure, any pre-existing medical conditions, smoking habits and BMI at the time of their first CVD. Participants were also interviewed and asked about their lifestyle at the time of their first CVD.

### Data analysis

Frequency distributions were used to summarise all the categorical demographic and health data. Responses for variables with ‘check all that apply’ options, such as those which captured information on co-morbidities or medications used, are ranked according to % calculated. Scale variables are described with summary statistics (means and standard deviations). Statistical tests of inference were used to estimate significant correlations (Chi-Square (χ^2^) tests of independence) between pairs of categorical variables, usually a demographic and a variable denoting categorical health status, or to test the significance of impacts of demographic and behavioural categorical independent variables on scale health data responses (*t* and ANOVA tests).

Risk Scores were obtained for the local study sample, using calculators from the existing risk models, namely the QRISK2 2015 Batch processor (Hippisley-Cox et al., 2007), the Framingham Risk Calculator and the ASSIGN Risk Calculator (Payne, 2012; Kannel, McGee & Gordon, 1976). Some of the participants had missing information on for variables which are used by the Framingham Risk calculator so scores for this model were obtained for only 727 total participants, including 485 non-CVD and 142 CVD individuals.

A receiver-operating characteristic (ROC) curve was used to quantify how well each model was able to discriminate between non-CVD and CVD participants. This was considered appropriate for comparing the three existing models because Area Under the Receiver-Operating Characteristic (AUROC) is commonly used as a measure of the overall performance of a diagnostic test and interpreted as the average value of sensitivity for all possible classification thresholds. A diagnostic test with an AUROC value greater than 0.5 is considered, therefore, better than relying on pure chance, and as having at least some ability to discriminate between subjects with and without a particular disease. Because sensitivity and specificity are independent of class prevalence, AUROC is also independent of any disparity in class (in this case CVD vs. non-CVD) sizes.

## Results

### Sample description

The participants in the study had an overall mean age of 46.0 years (±12.8)—Table 1. Slightly more than half (53%) of the sample was female, most (81%) had a secondary or higher-level education, and almost half (48%) of the sample was single. There was a relatively even distribution among the racial groups of Afro-Trinbagonians (35%), Indo-Trinbagonian (32%), and Mixed-Trinbagonian (31%). Most were non-smokers (83%), with the remaining 17% being either a smoker or an ex-smoke. The TT2015 sample had a mean BMI of 26, with 53% classed as overweight/obese.

**Table 1:** Distribution of the major characteristics measured among the non-CVD* and CVD participants for the sample (*n* = 778).

Variable and categories (acceptable levels)	Total %/mean (SD)	Non-CVD % freq./mean (SD)	CVD % Freq./mean (SD)	*p*
Age (years)	46 (12.8)	35 (14.1)	56 (11.5)	0.000
Age (%)				0.000
18–25	21.1	96.2	3.8	
26–41	29.7	90.1	9.9	
42–56	24.1	54.1	45.9	
57–75	25.1	27.5	72.5	
Sex (%)				0.001
Male	47.3	61.2	38.8	
Female	52.7	73.1	26.9	
Ethnic group (%)				0.000
African	34.6	84.8	15.2	
East Indian	32.5	52.6	47.4	
Mixed	31.0	64.8	35.2	
Other	2.0	46.7	53.3	
Education level (%)				0.305
None/Primary	19.8	70.4	29.6	
Secondary	49.6	65.0	35.0	
Tertiary	30.5	70.1	29.9	
Marital status (%)				0.000
Single	47.7	86.2	13.8	
Married/Common-law	41.0	48.0	52.0	
Divorced/Separated/Widowed	11.3	53.6	46.4	
Cigarette smoking (%)				0.000
Never	82.9	71.2	28.8	
Ex-smoker	8.9	9.8	90.2	
Current smoker	8.2	75.8	24.2	
BMI (kg/m^2^) (18.5–24.9)	26 (5.9)	26 (6.2)	26 (5.6)	0.130
WHR (cm/cm)	0.86 (0.10)	0.83 (0.09)	0.94 (0.07)	0.000
Resting B.P. (mm Hg)				
Mean systolic (90–140)	124 (22.4)	121 (19.9)	126 (24.8)	0.000
Mean diastolic (60–90)	82 (13.1)	82 (12.7)	82 (13.4)	0.995
Lipid profile (mg/dL)				
Total cholesterol (<200)	166 (46.5)	151 (37.0)	181 (56.0)	0.000
LDL (<100)	91 (25.1)	78 (3.4)	104 (46.8)	0.000
HDL (>40 )	47.5 (18.2)	49 (17.8)	46 (18.5)	0.066
Triglycerides (<150 )	130 (88.4)	120 (82.9)	139 (93.9)	0.015
Co-morbidities (%)				
High cholesterol	25.9	23.4	76.6	0.000
High Blood Pressure (HBP)	21.1	6.7	93.3	0.000
Diabetes	18.1	29.3	70.7	0.000
Atrial fibrillation	8.7	7.7	92.3	0.000
Chronic kidney disease	1.4	9.1	90.9	0.000
Rheumatoid arthritis	1.2	11.1	88.9	0.000
Family history of (%)				
Diabetes	63.1	64.2	35.8	0.005
HBP	62.5	63	37	0.000
CVDs	38.6	37.8	62.2	0.000
High cholesterol	24.4	64.7	35.3	0.152

**Note:**

* Cardiovascular disease.

High blood pressure, which is the second most prevalent (21.1%) co-morbidity reported for this sample and several of the less prevalent conditions, such as high atrial fibrillation, chronic kidney disease and rheumatoid arthritis were almost exclusively (89 to 93% of the total prevalence reported) in the CVD sub-group (Table 1). Other adverse health conditions, like diabetes (18.1%) and high cholesterol (25.9%), which are among the most prevalent for this sample, were also mainly in the CVD sub-group. The effect is even more marked when considering that the non-CVD sub-group was twice the size of the CVD sub-group.

### Comparison of CVD risk scores from the three models

The percentage distribution of low, moderate, and high risk scores, for the CVD (diagnosed CVD event) and non-CVD (no diagnosed CVD event) sub-groups by the three models, are given in Table 2 and the mean scores in Table 3. All the models correctly classified the group of non-CVD participants as between 87% (Framingham) and 92% (QRISK^®^2) low risk. However, for persons who have had a CVD event, the three risk models were only able to identify 33% (ASSIGN) to 50% (Framingham) of CVD patients as having a high 10-year risk of a CVD event, while 24% (ASSIGN) to 31%% (QRISK^®^2) were misclassified as being at moderate or intermediate risk. The QRISK^®^2 and Framingham models misclassified 21% and 22% respectively of the CVD group as low risk, but the ASSIGN categorised as many as 43% of the CVD patients as being at low risk of CVD. For each model, though, the mean scores for the CVD and non-CVD sub-groups were still estimated as significantly different (*t*-tests, *p* = 0.000), in spite of the apparently high misclassifications for the CVD group. This could happen even with a small effect size if the standard error, a measure of the variability in scores within each sub-group, is low. This would lead to a low pooled estimate for the standard error and a *t* statistic, high enough to be significant.

**Table 2:** Percentage distribution of scores categorised by the ASSIGN, Framingham and QRISK^®^2 models, for non-CVD* and CVD sample.

CVD status	Risk model	% Frequency		
		Low risk score	Moderate risk score	High risk score
		<10%	10–20%	>20%
Non-CVD	ASSIGN	90	6	4
	Framingham	87	8	5
	QRISK^®^2	92	5	3
CVD	ASSIGN	43	24	33
	Framingham	22	28	50
	QRISK^®^2	21	31	48

**Note:**

* CVD, cardiovascular disease.

**Table 3:** Mean scores for each risk model for non-CVD* and CVD groups in the sample.

Risk model	NON-CVD		CVD	
	*n*	Mean (SD)	*n*	Mean (SD)
ASSIGN Risk Score	526	3.7 (6.7)	252	16.6 (14.9)
Framingham Risk Score	485	3.9 (7.2)	142	22.8 (14.8)
QRISK^®^2 Risk score	526	2.7 (6.6)	252	23.5 (17.4)

**Note:**

* CVD, cardiovascular disease.

### ROC of models

**Figure 2:**
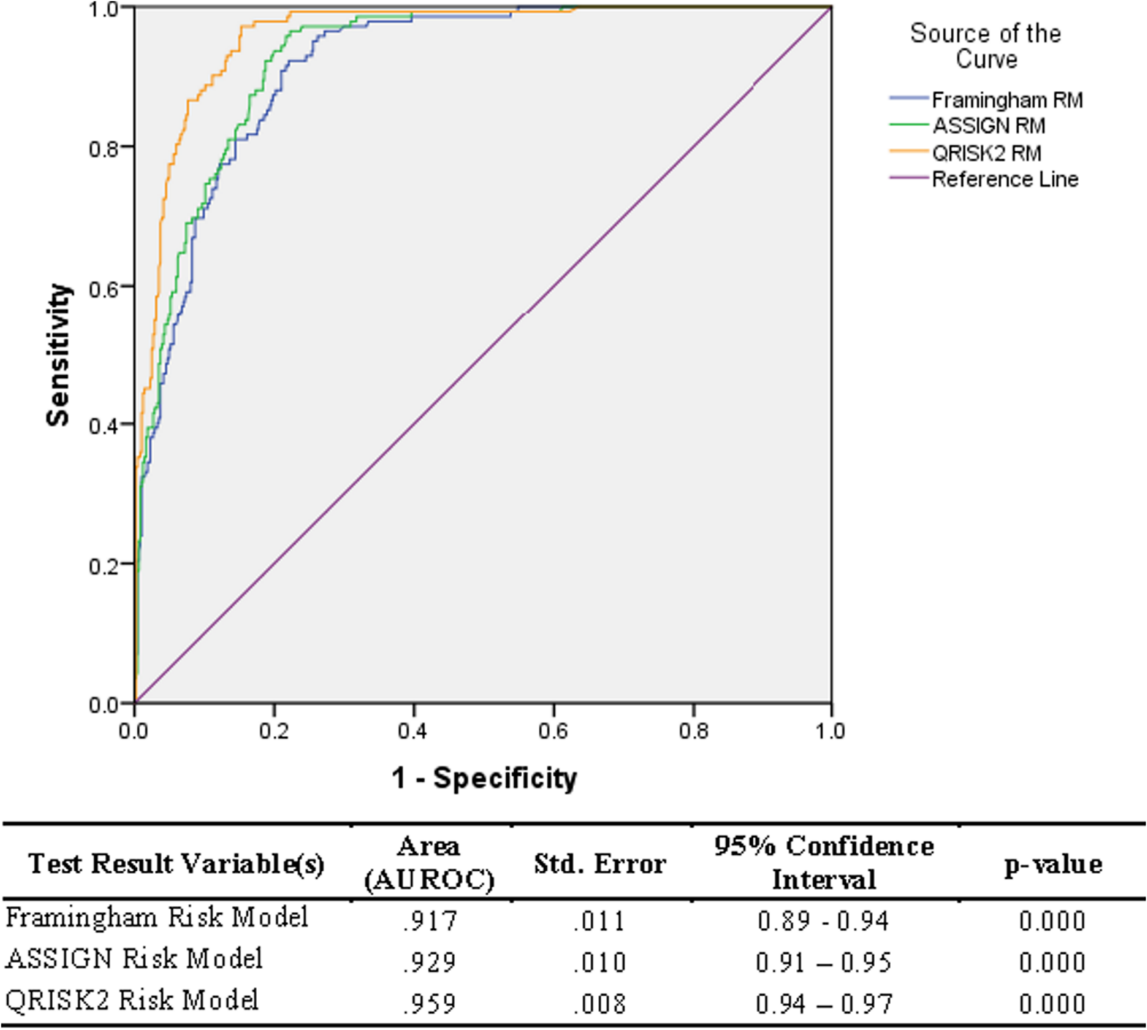
Relative Operating Characteristic (ROC) plot for three established CVD risk prediction models in the Trinidad and Tobago population.

The risk scores from the three models were found to be significantly different (*p*-value = 0.001), as estimated by one-way ANOVA tests for each of the non-CVD and CVD groups. Overall, the QRISK^®^2 risk prediction model (AUROC = 0.96) performed the best compared to the ASSIGN (AUROC = 0.93) and the Framingham risk prediction model (AUROC= 0.92)—Fig. 2.

However, as illustrated in Table 2, there was a low success rate in identifying the CVD sub-group scores as high risk with only 48% of CVD identified by the QRISK^®^2 model, 50% identified by the Framingham (50%) model, and 43% by ASSIGN, as previously stated.

### Classification efficiency of the risk models

**Figure 3:**
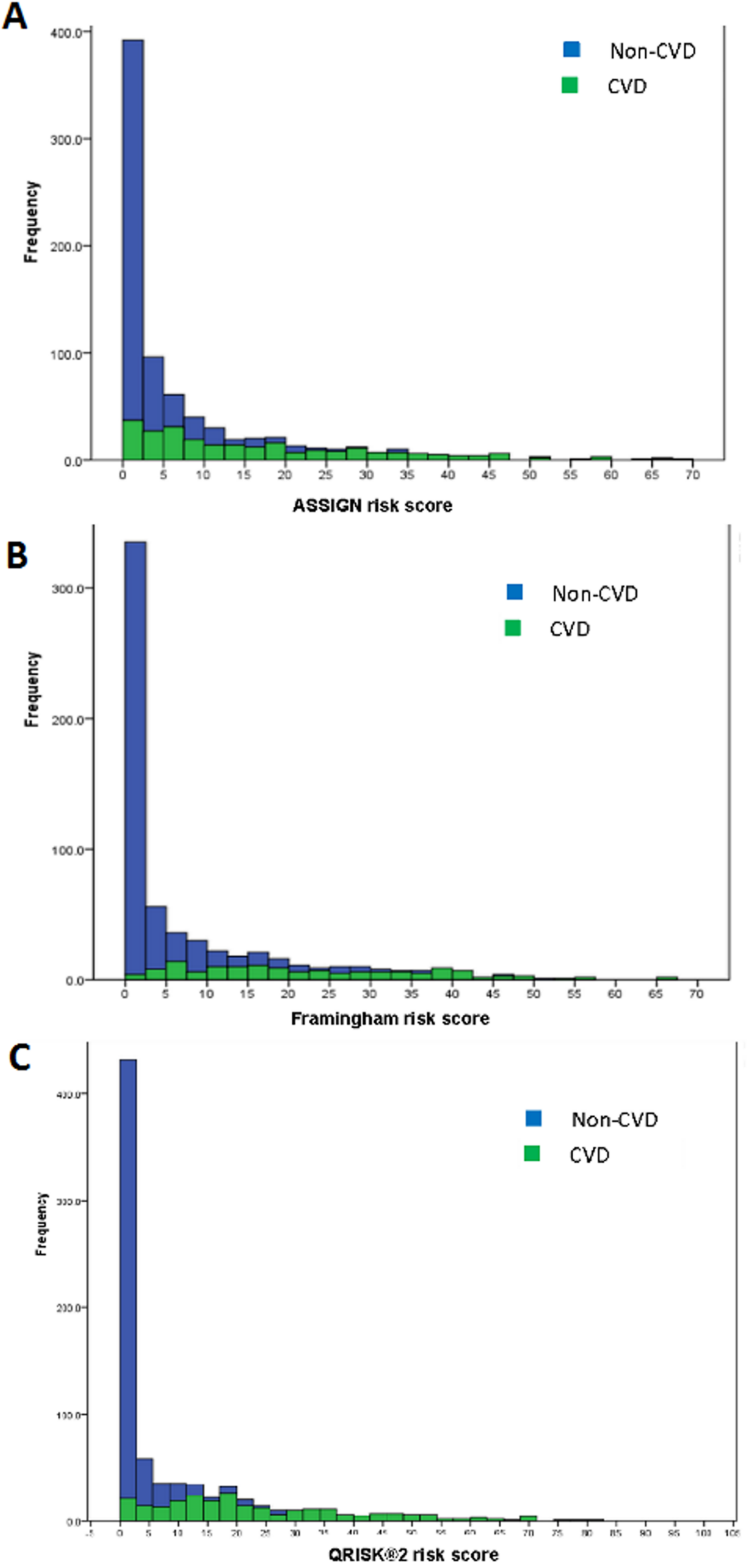
Stacked histograms for the risk scores from the ASSIGN (A), Framingham (B), and QRISK^®^2 (C) models for non-CVD and CVD participants in the TT2015 sample.

Stacked histograms of the risk scores for all three models are shown in Fig. 3. From the axes in these graphs, three ranges of scores were extracted—that in which the individuals were almost exclusively non-CVD, another in which most were CVD patients, and a third range of scores in which there was considerable overlap in the estimated scores for individuals from the two groups. This last mentioned range was termed the non-differentiating or misclassification range. Cross-tabulations of these ranges with actual CVD status was done for each risk model and led to the information in Tables 4 and 5. The information for the non-differentiating range in Table 4 is the % of the scores for the total sample which falls in that range.

**Table 4:** Sensitivity, specificity, and non-differentiating % for ASSIGN, Framingham and QRISK^®^2 risk models.

Risk model	Non-CVD		Non-differentiating		CVD	
	Range	Specificity	Range	%	Range	Sensitivity
ASSIGN RS	0.03–4.99	0.80	5.00–12.50	16.8	12.51–69.39	0.50
Framingham RS	0.00–9.99	0.87	10.00–22.50	14.0	22.51–65.10	0.45
QRISK^®^2 RS	0.00–4.99	0.86	5.00–15.00	13.5	15.01–81.04	0.62

All the criteria in Table 5—sensitivity (% of Total CVD correctly predicted), specificity (% Total non-CVD correctly predicted), positive predictability (% of those individuals predicted as CVD who are actually CVD) and negative predictability (% of those individuals predicted as non-CVD who are really non-CVD), total % sample in the non-differentiating range and the probability of finding CVD patients in this range—supported the efficacy of the classification. The QRISK^®^2 performed the best out of the three models, having the he smallest percentage of individuals in the non-differentiating range and almost the smallest relative risk of a CVD patient’s score falling into this range. Hence, if an individual received a QRISK^®^2 score of 5 to 15 (i.e. in the non-differentiating range), then that individual would be 2.6 times more likely to be a CVD patient than a non-CVD participant (Table 5).

**Table 5:** Classification characteristics for ASSIGN, Framingham and QRISK2 models for the sample.

Characteristic	ASSIGN	Framingham	QRISK^®^2
Sensitivity	49.6	44.7	62.1
Specificity	80.3	87.4	86.4
Positive predictability	77.9	77.8	87.0
Negative predictability	87.1	92.9	92.3
Non-differentiating risk score range	5.00–12.50	10.00–22.50	5.00–15.00
Total % population in non-differentiating range	16.84	14.04	13.50
Relative risk of CVD being in non-differentiating range	1.99	3.74	2.58

By comparison, the Framingham and ASSIGN risk models showed mixed results with this sample. ASSIGN was more sensitive but the Framingham had a higher specificity. They both showed comparable positive predictabilities but the Framingham had the better negative predictability. The other measures were also mixed. The ASSIGN model had the largest percentage of individuals in the non-differentiating range but most of these were non-CVD individuals since it has the lowest relative risk of a CVD individual’s score being in this range.

For the sample, all three models correctly predicted less than 62% of cases (CVD participants) with positive predictabilities ranging from 78% (ASSIGN and Framingham) to 87% (QRISK^®^2). Further, the non-differentiating ranges for the ASSIGN, Framingham, and QRISK^®^2 scores were all 2 or more times as likely to include CVD participants.

### Risk models and ethnicity

Examining each risk model by ethnicity, the QRISK^®^2 model was able to improve its sensitivity from 62% for the entire sample to 72% for Indo-Trinbagonians (Table 6). The differences were not as marked for the other two ethnic groups. Whereas the Framingham model showed poorer sensitivity, particularly for the mixed-Trinbagonians, it was either the best (for Indo- and Mixed- sub-groups) or second to the QRISK^®^2 model (for Afro-Trinbagonians) in correctly identifying non-CVD individuals (specificity).

**Table 6:** Discrimination power of each of the three models tested in this study by ethnicity.

Ethnicity	Category	Established CVD risk models		
		ASSIGN	Framingham	QRISK^®^2
Afro-Trinbagonian (*n* = 269)	Correct non-CVD (%)	81.2	87.9	90.6
	Non-CVD in non-differentiating range (% total)	13.0	7.0	6.7
	CVD in non-differentiating range (% total)	20.0	33.3	30.0
	Correct CVD (%)	50.0	50.0	52.5
Indo-Trinbagonian (*n* = 253)	Correct non-CVD (%)	80.0	88.3	81.5
	Non-CVD in non-differentiating range (% total)	13.1	10.0	10.0
	CVD in non-differentiating range (% total)	23.9	31.1	12.8
	Correct CVD (%)	49.6	45.9	71.8
Mixed-Trinbagonian (*n* = 241)	Correct non-CVD (%)	80.4	87.5	85.6
	Non-CVD in non-differentiating range (% total)	11.1	9.6	10.5
	CVD in non-differentiating range (% total)	31.3	36.5	33.7
	Correct CVD (%)	50.6	38.5	54.2

The total number of individuals and the distribution percentages between CVD and non-CVD individuals in the non-differentiating ranges were almost the same for the three models when classifying mixed-Trinbagonians. For the Indo-Trinbagonian sub-group, however, the QRISK^®^2 model was markedly better than the other two, but the ASSIGN model, which overall had the largest % (16.8) of individuals from the total sample in the non-differentiating range, had the smallest total % (33) of Afro-Trinbagonians and the smallest % (20) of that ethnic group with CVD in this range. It is the only with this ethnic group that ASSIGN performed the best of the three models (Table 6).

## Discussion

Scores from all three models (ASSIGN, Framingham, and QRISK^®^2) at first glance showed good discrimination, between the CVD participants and the non-CVD participants (AUROC 0.917 to 0.959)—Fig. 2. However, the scores from the three models were still significantly different from each other. Similarly, comparison by ANOVA of the risk scores for the three models show them to be significantly different as well, corroborating the differences seen in the AUROC values.

From the literature, the Framingham and QRISK models have correctly predicted approximately 70% of cases, with a high ratio of false-positive predictions to true predictions (Marsh, 2011). Studies on a 1.8 million person sample, done in England (Van Staa et al., 2014; Tillin et al., 2014) and on a sample of 1400 Czech men (Reissigova & Zvarova, 2007) showed underestimation of the scores for individuals with CVD using these models. Other studies found that the Framingham and QRISK^®^2 models were over-predicting risk, even in the samples which included the two ethnic sub-groupings of African Caribbeans and South Asians (Tillin et al., 2014; Rabanal et al., 2018).

In this study, the ASSIGN, Framingham, and QRISK^®^2 models underestimated scores for persons with CVD in our sample (Sensitivity = 50 to 62%), but were very effective at estimating scores for the non-CVD persons (Specificity = 80–87%). The AUROC values showed excellent discrimination ability for the three models, but this can be accounted for by the smaller CVD sample (252 persons) compared to non-CVD (526 persons). Hence, the overall assessment of discrimination (using AUROC values) was skewed by the non-CVD sub-group’s results. However, a high sensitivity is clearly important and preferable to high specificity when a test is used to identify a serious but treatable non-communicable disease such as a CVD (Lalkhen & McCluskey, 2008). Studies show that presence-absence predictions exclude AUROC plotting and, thus, AUROC is not always the technique which should be used for evaluating accuracies of the prediction estimates (Shabani, Kumar & Ahmadi, 2018). Thus, the high values of AUROC for each model is no guarantee of classification accuracy. Siontis, Tzoulaki, Siontis, Ioannidis (Siontis et al., 2012) reported, in their comparison of established risk prediction models for CVD which uses the AUROC metric, that the QRISK^®^2 model also outperformed Framingham and ASSIGN models with their sample.

Some comorbidities with CVD status, such as high blood pressure, high atrial fibrillation, chronic kidney disease, and rheumatoid arthritis, although overall not very prevalent, except for high blood pressure, were reported almost exclusively (89 to 93% of the total) by individuals in the CVD sub-group (Table 1). Most of the individuals suffering with diabetes and high cholesterol, which were among the three most prevalent comorbidities, were also mainly (71 to 77% of all reported cases) in the CVD sub-group. The effect is even more marked when considering that the non-CVD sub-group is twice the size of the CVD sub-group. CVD risk factors such as age, sex, ethnicity, presence of High Blood Pressure, BMI etc. have all been used by risk prediction models to estimate a person’s likelihood of having a CVD event (Larifla et al., 2014; Davis et al., 2010). Arithmetic weights for each risk factor are used to determine that risk factor’s contribution towards an overall CVD risk (Davis et al., 2010). These weights, based on established population data, which are used to calculate a risk score, which is used in further analyses (Hippisley-Cox et al., 2008; Sullivan, Massaro & D’Agostino, 2004; Woodward, Brindle & Tunstall-Pedoe, 2007).

The identification of the new non-differentiating ranges of scores, attributable to both non-CVD and CVD participants, for the three CVD risk models, provided additional insights beyond those standard classification metrics (Table 6). For all three models, the relative risk of a CVD versus a non-CVD participant being in the non-differentiating range was 2 or higher. Hence, the overall sensitivity and the relative risk of finding a CVD individual in the non-differentiating range were together far more relevant than any other metrics in evaluating the usefulness of the model for estimating CVD risk.

One of the questions of interest in this study of a multi-ethnic sample was whether ethnicity confounded the classification power of the models. Distinctive classification patterns were observed for the different ethnicities with all the models (Table 6). In this multi-ethnic sample, it was observed that different models worked best in classifying different ethnic sub-groups. The QRISK^®^2 model was by far the most successful at identifying Indo-Trinbagonians with CVD and it also had the smallest number of total participants and of those with CVD in the non-differentiating range for this ethnic sub-group. In fact, this model is the most sensitive for all ethnic groups but it does not perform as well in the non-differentiating range for the Afro- and Mixed Trinbagonian groups as it does for the Indo-Trinbagonians. For the subsets of Afro- and Mixed-Trinbagonians, the ASSIGN Model performed the best at identifying CVD participants in the non-differentiating range (Table 6). The Framingham model was the worst performer for any of the ethnic sub-groups.

From the performance metrics for classification given in Table 5, the QRISK^®^2 showed the best sensitivity and positive predictability and very close to the best specificity and negative predictability as a classifier model for this sample of the Trinidad and Tobago population. In this study, though, the other characteristics in Table 5 add some interesting insights. Ideally, if the risk scores are to be used in a functional manner, then the number of individuals who fall into the non-differentiating range should be minimal. It is also preferable to have fewer CVD patients’ scores in this range, since some physicians may prefer to have further confirmatory tests for patients who fall in this range before diagnosing and treating the disease.

It is anticipated that the findings from this study will prevent less misclassification of persons at risk for CVD, by using the most suitable model for the ethnicity of the patient, as well as inform physicians and policymakers about the accuracy of established systems for Caribbean populations. Population-specific and evidence-based health information systems are recommended for Caribbean populations to start understanding and lowering the epidemic of heart disease in this region.

## Conclusions

The AUROC values showed a goodness of fit between 90% and 94% for the three established models (i.e. ASSIGN, Framingham and QRISK^®^2), when discriminating between CVD and non-CVD participants. However, the sensitivity of the models showed that CVD patients were correctly classified by all three models for only 50–62% of the total cases. Generally, AUROC should be used cautiously when determining the goodness of fit for risk classification models. Sensitivity and specificity values were the more useful classifiers in this study. Ethnicity does affect the classification performance of all the models. The QRISK^®^2 model was the overall best performer when applying the metrics of sensitivity, specificity, and positive and negative predictabilities to the total sample, but it was also clearly the best classification model for Indo-Trinbagonians and marginally the best for Mixed-Trinbagonians.

## Supplemental Information

10.7717/peerj.8232/supp-1 Supplemental Information 1 Differences between 3 CVD risk prediction models. Source: D’Agostino et al. (2008); Hippisley-Cox et al. (2007); Hugh Tunstall-Pedoe (2011).

10.7717/peerj.8232/supp-2 Supplemental Information 2 Risk predictors used for developing a Cardiovascular Risk Score in the Framingham, ASSIGN and QRISK2 models.

10.7717/peerj.8232/supp-3 Supplemental Information 3 Percentage distribution of persons categorised by three established Risk Models into different risk levels for non-CVD and CVD groups from the TT2015 study on a Trinidad and Tobago sample (*n* = 778).

10.7717/peerj.8232/supp-4 Supplemental Information 4 Comparison of ASSIGN, Framingham, and QRISK^®^2 CVD risk models based on the CVD, Non-CVD and non-differentiated ranges.

## Additional Information and Declarations

### Data Availability

The following information was supplied regarding data availability: Data is available at Figshare: Hosein, Amalia (2019): Data file for CVD established models paper TT2015 casecontrol.csv. figshare. Dataset. DOI 10.6084/m9.figshare.7752440.v1.
