# Supplementary material for: Evaluating Cardiovascular Disease (CVD) risk scores for participants with known CVD and non-CVD in a multiracial/ethnic Caribbean sample
Source: PeerJ. 2020 Mar 9;8:e8232. doi: 10.7717/peerj.8232 (PMC7067186; doi:10.7717/peerj.8232)
Supplement: Supplemental Information 1 — Source: D’Agostino et al. (2008); Hippisley-Cox et al. (2007); Hugh Tunstall-Pedoe (2011). [file peerj-08-8232-s001.docx]

Supplemental Table 1: Differences between 3 CVD risk prediction models

|  | FRAM | ASSIGN | QRISK2 |
| --- | --- | --- | --- |
| Age | Yes | Yes | Yes |
| Sex | Yes | Yes | Yes |
| Cholesterol levels | Yes | Yes | Yes |
| Smoking | Yes | Yes | Yes |
| BP treatment | Yes | No | Yes |
| Diabetes | Yes | Yes | Yes |
| Family History | No | Yes | Yes |
| Social deprivation | No | Yes | Yes |
| Ethnicity | No | No | Yes |
| Reproducibility | Yes | Yes | Yes |
| Generalisability | - | - | Yes |
| Statistical validity | Yes | Yes | Yes |
| Face validity | Yes | No | Yes |

Source: [D’Agostino et al. (2008)](#_ENREF_33); [Hippisley-Cox et al. (2007)](#_ENREF_56); [Hugh Tunstall-Pedoe (2011)](#_ENREF_132)
