# Supplementary material for: Evaluating Cardiovascular Disease (CVD) risk scores for participants with known CVD and non-CVD in a multiracial/ethnic Caribbean sample
Source: PeerJ. 2020 Mar 9;8:e8232. doi: 10.7717/peerj.8232 (PMC7067186; doi:10.7717/peerj.8232)
Supplement: Supplemental Information 2 [file peerj-08-8232-s002.docx]

Supplemental Table 2: Risk predictors used for developing a Cardiovascular Risk Score in the Framingham, ASSIGN and QRISK2 models

| PREDICTOR | Framingham | ASSIGN | QRISK2 |
| --- | --- | --- | --- |
| Age (years) | ✓ | ✓ | ✓ |
| Presence of HBP |  |  | ✓ |
| Family history of CVD | ✓ | ✓ | ✓ |
| Presence of Atrial Fibrillation |  |  | ✓ |
| Sex | ✓ | ✓ | ✓ |
| Smoking | ✓ | ✓ | ✓ |
| TC/HDL | ✓ | ✓ | ✓ |
| Diabetic | ✓ | ✓ | ✓ |
| Diastolic BP |  |  | ✓ |
| No. of cigarettes/day |  | ✓ | ✓ |
| Presence of Left Ventricular Hypertrophy |  | ✓ |  |
| Social deprivation† |  | ✓ | ✓ |
| Systolic BP | ✓ | ✓ | ✓ |
| BMI |  |  | ✓ |
| Presence of Rheumatoid arthritis |  |  | ✓ |
| Presence of chronic kidney disease |  |  | ✓ |
| Ethnicity |  |  | ✓ |
