# Supplementary figures and images for: Evaluating Cardiovascular Disease (CVD) risk scores for participants with known CVD and non-CVD in a multiracial/ethnic Caribbean sample
Source: PeerJ. 2020 Mar 9;8:e8232. doi: 10.7717/peerj.8232 (PMC7067186; doi:10.7717/peerj.8232)

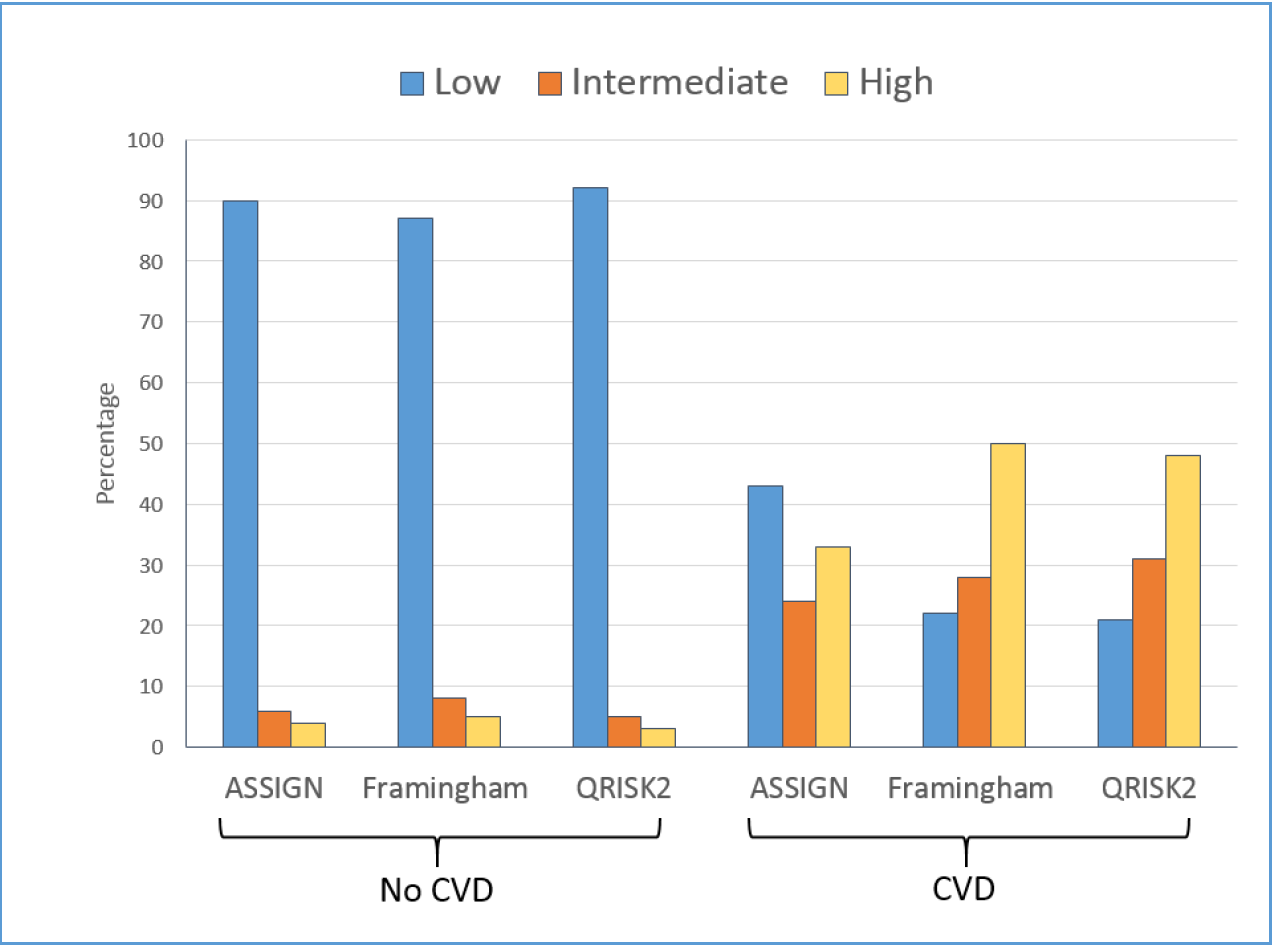

Supplement: Supplemental Information 3 [file peerj-08-8232-s003.png]

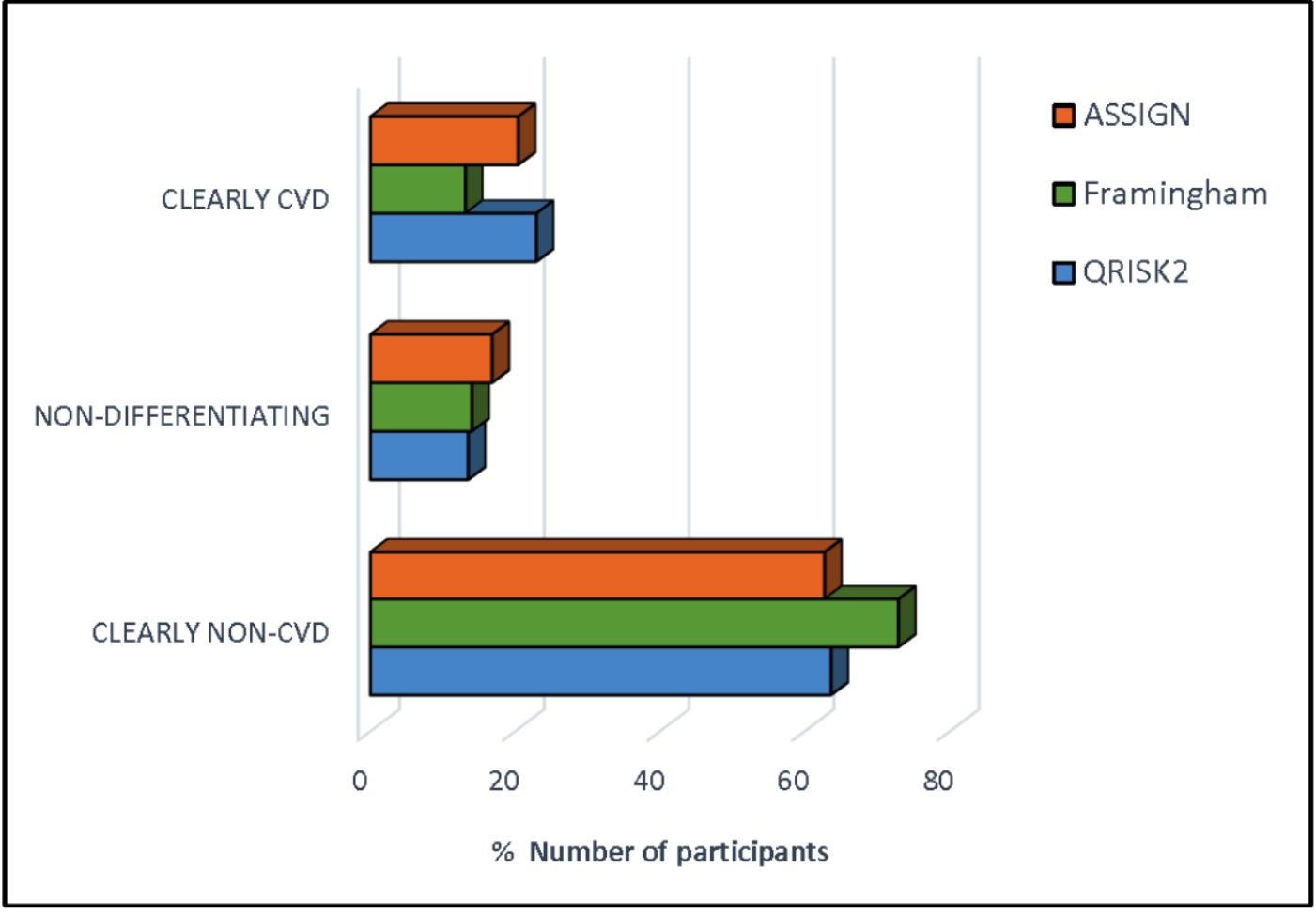

Supplement: Supplemental Information 4 [file peerj-08-8232-s004.png]
